# From plant pangenome assemblies to operational graph references

**DOI:** 10.3389/fpls.2026.1997114

**Published:** 2026-10-06

**Authors:** Nunzio D’Agostino

**Affiliations:** Department of Agricultural Sciences, University of Naples Federico II, Naples, Italy

**Keywords:** crop genomics, genomic infrastructure, graph genomes, plant pangenome, reference bias, structural variation (SV), variation graphs

## Introduction

Plant pangenomes have established that a single linear assembly cannot represent the sequence and structural diversity of a species.

Plant pangenome studies have revealed how genomic diversity contributes to domestication, adaptation, and agronomic traits. In *Brassica oleracea*, early analyses identified extensive gene presence–absence variation involving genes implicated in disease resistance, flowering time, and specialized metabolism (Golicz et al., 2016). Sunflower pangenome analysis subsequently showed how introgression from wild relatives altered cultivated gene content and contributed to disease resistance (Hübner et al., 2019), while comparisons of 26 maize genomes revealed the extent of genomic diversity overlooked by a single reference (Hufford et al., 2021). In rice, a graph pangenome constructed from 33 genetically diverse accessions revealed extensive structural and gene copy-number variation and enabled the identification of trait-associated variants missed by SNP-based analyses using a single reference genome (Qin et al., 2021). In tomato, initial work recovered genes missing from the reference assembly and identified a rare allele associated with fruit flavor (Gao et al., 2019). A subsequent tomato graph pangenome extended these advances by incorporating structural variation into analyses of trait heritability and breeding prediction (Zhou et al., 2022). More recently, an eggplant study integrated 40 chromosome-level assemblies into two graph pangenomes and combined these resources with phenotyping of 368 accessions to identify variants underlying prickliness, Fusarium wilt resistance, and fruit metabolite composition (Gaccione et al., 2025). Together, these findings demonstrate how pangenomes connect genomic diversity to biological function and crop improvement.

Translating these discoveries into routine analysis requires a robust technical framework for representing, accessing, and interpreting genomic variation. Graph pangenomes encode shared and alternative sequences and their relationships within a common reference structure, supporting analyses beyond the scope of a single linear genome. Their practical use depends on reliable graph construction, graph-aware genotyping, and annotation methods that accommodate the complexity of plant genomes (Schreiber et al., 2024; Bao and Weigel, 2025). Beyond these analytical methods, consistent resource development and exchange require common identifiers, metadata, quality control, evaluation, and data-sharing practices, as proposed by recent standards initiatives (Heuermann et al., 2025; Kopalli et al., 2025). Connecting these resources with functional panomics further requires reproducible workflows that support their use across datasets and research communities (Cochetel and Cantu, 2026).

A narrower infrastructure gap nevertheless remains: a collection of assemblies is not an operational pangenome, and a downloadable graph file is not necessarily a usable graph reference. I argue that mature plant pangenome projects should release an operational, versioned graph-reference package as a standard deliverable. Such a package combines graph topology and biological paths with projected annotations, coordinate mappings, task-specific indexes, validation datasets, reproducible workflows, release histories, and governance (**Figure 1**). This distinction shifts the discussion from whether graphs are useful, which is already well established, to what must accompany a graph before a research community can use it routinely.

**Figure 1:**
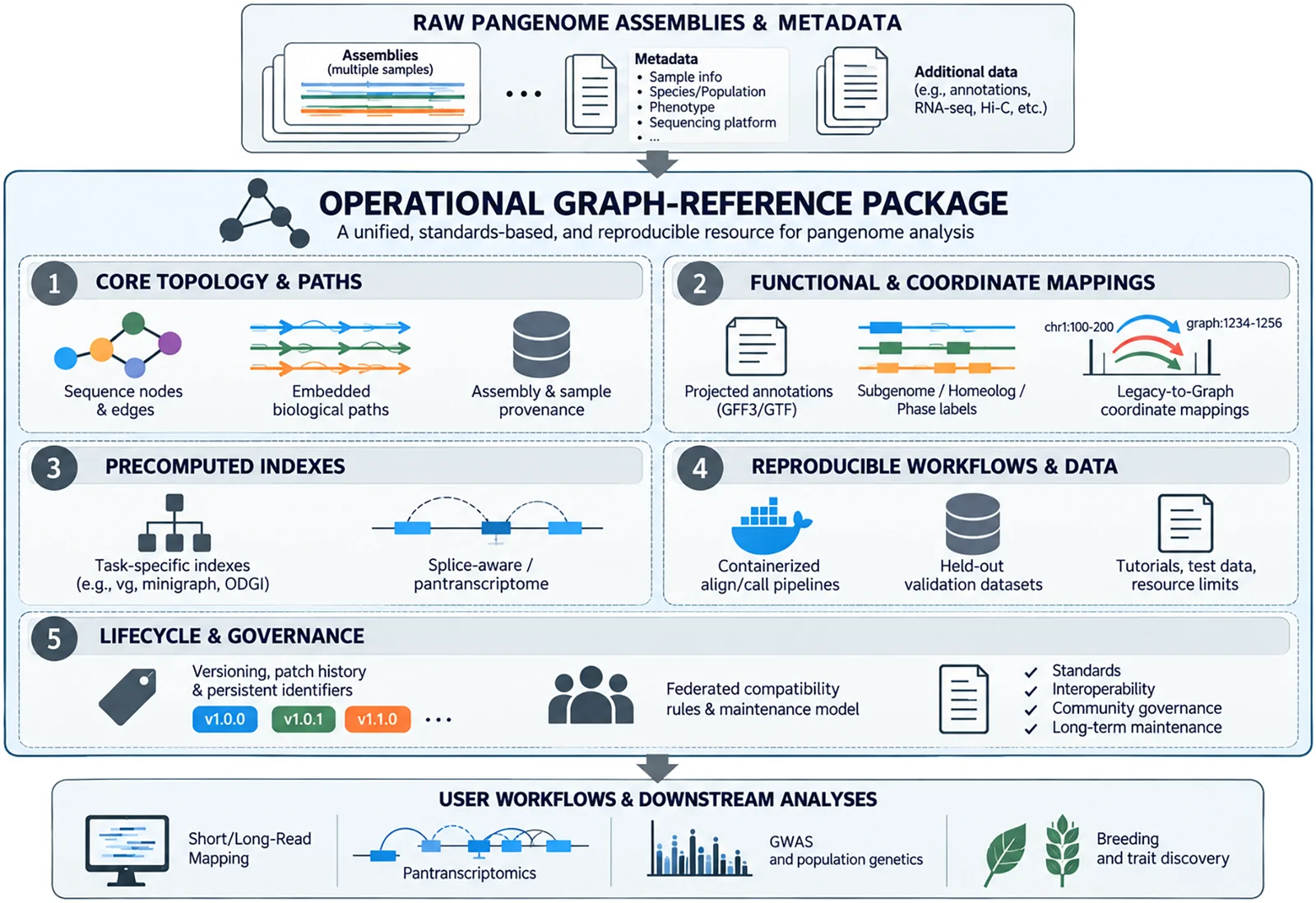
Conceptual framework for an operational graph-reference package for pangenome analysis. Raw pangenome assemblies and associated metadata are integrated into a unified graph-reference resource comprising five major components: (1) core graph topology and embedded biological paths, with assembly and sample provenance; (2) projected functional annotations, subgenome/homeolog/phase labels, and legacy-to-graph coordinate mappings; (3) precomputed task-specific and splice-aware indexes; (4) reproducible, containerized analysis workflows, validation datasets, tutorials, and test data; and (5) lifecycle and governance mechanisms supporting versioning, persistent identifiers, interoperability, and long-term maintenance. Together, these components provide a reproducible foundation for downstream applications including short- and long-read mapping, pantranscriptomics, genome-wide association studies (GWAS), population genetics, breeding, and trait discovery.

## Minimum requirements and validation

The baseline is the familiar single-genome release. An annotated reference distributed through NCBI or Ensembl typically makes available genomic FASTA, gene models in GFF3 or GTF, and transcript, coding-sequence, and peptide FASTA files; repeat or transposable-element annotations may be supplied separately. The central organizing principle of such releases is shared sequence identifiers and coordinates, allowing users to connect reads, genes, and variants within a documented assembly version. A graph pangenome release must preserve these basic functions while accommodating multiple assemblies, alternative sequences, and the paths that represent individual genomes or haplotypes. Unlike a single-genome package, it must document both the relationships among those sequences and how annotations and coordinates translate across them. Its foundation should comprise quality-controlled, chromosome-scale assemblies, stable accession metadata, and adequate representation of the diversity for which the resource is intended. Its graph release should include interoperable topology and embedded paths, assembly and sample provenance, projected annotations, legacy-coordinate mappings, precomputed indexes, containerized workflows, tutorials, test data, and explicit computing requirements. Canonical biological content should remain separable from software-specific indexes so that communities can support evolving toolchains such as vg, minigraph, minigraph-cactus, PGGB, and ODGI (Garrison et al., 2018; Li et al., 2020; Guarracino et al., 2022; Hickey et al., 2024; Garrison et al., 2024).

Validation should accompany publication rather than be deferred to downstream users. Releases should report graph growth as genomes are added, sequence and path completeness, topological complexity, mapping accuracy, reference bias, variant-genotyping performance, and computational cost. Held-out accessions or haplotypes are preferable because evaluation only on genomes used for construction overstates generalizability. Benchmarks should include repetitive and structurally complex regions and, where relevant, polyploid-specific tests of homeolog assignment, subgenome labels, phasing, and collapsed sequence (Kopalli et al., 2025).

Graph composition also requires scrutiny. A panel dominated by elite or closely related material can reproduce sampling bias even when its graph is technically complete. Conversely, adding increasingly divergent genomes may improve representation while increasing topology, index size, and ambiguous mapping. Graph references should therefore state their sampling purpose and limitations. They can reduce biological reference bias while increasing computational and training demands; those trade-offs should be measured rather than obscured.

## From assemblies to an operational reference

Assemblies describe diversity but leave each user to reconstruct relationships among genomes. Whole-genome alignment, repeat handling, variant normalization, path naming, and coordinate translation are consequential choices. Repeating them independently makes analyses inconsistent and unnecessarily expensive. Variant call files reduce part of this burden, but they remain anchored to a privileged reference and can represent nested, multiallelic, or overlapping structural variants awkwardly. Alleles and genes absent from that reference may be difficult to map, genotype, or annotate consistently.

A sequence graph addresses the representation problem by encoding sequences as nodes, alternative adjacencies as edges, and chromosomes or haplotypes as paths (Paten et al., 2017). To make this representation reproducible, releases should document a parameter-complete construction pipeline and rules for representing uncertainty and collapsed sequence. Gene, repeat, and regulatory annotations should be linked to the relevant paths. These choices determine how biological content connects to supported analytical tasks.

## Keeping track of chromosome copies in polyploid crops

Polyploid crops have several similar chromosome copies. Graph-reference packages must distinguish copies within the same subgenome (homologs) from related chromosomes inherited from different ancestral genomes (homeologs). This distinction helps avoid errors when analysing variation, gene expression, and gene dosage in wheat, oat, sugarcane, and polyploid brassicas (Walkowiak et al., 2020; Song et al., 2020; Kamal et al., 2022; Healey et al., 2024). Each graph path should have a stable identifier linked to a machine-readable registry recording species, accession, assembly version, subgenome, chromosome, haplotype or copy, and phase-block identifier. Readable aliases, such as accession|assembly|A|1A|hap1, can help users recognize these attributes, but must follow the naming rules of the supplied tools. Subgenome labels should follow established species conventions, such as A, B, and D in wheat. Uncertain ancestry or copy identity should be recorded explicitly. Releases should explain the extent and evidence of phasing—the assignment of sequences to individual chromosome copies. A locally phased region should not be presented as a chromosome-long haplotype without supporting evidence. Suspected switches between copies, regions where copies have been merged during assembly, and unresolved regions should be flagged. Homeolog assignment should combine sequence similarity with conserved gene order and ancestral evidence where available. Where segments have been exchanged between subgenomes, ancestry should be recorded for those segments rather than inferred solely from the chromosome name.

When transferring coordinates between references, tools should first seek the corresponding region in the same subgenome, while accounting for known exchanges between subgenomes. If several locations are plausible, the output should report the alternatives and their assignment confidence.

## RNA-sequencing exposes the implementation gap

RNA-seq data from diverse varieties or accessions are still commonly analysed by aligning all reads to a single reference genome. Transcripts carrying divergent alleles, accession-specific exons, or genes absent from that reference may map less efficiently, be misassigned, or fail to map, biasing transcript detection, expression estimates, and allele-specific expression. Genotype-specific references improve transcript quantification in several crops, and spliced pangenome graphs can support haplotype-aware pantranscriptome analysis (Sibbesen et al., 2023; Cochetel and Cantu, 2026).

In polyploid crops, highly similar transcripts from different chromosome copies make read mapping particularly challenging (Aufiero et al., 2024). Variation graphs can help by representing homologous and homeologous sequences together, allowing reads to be compared against alternative copies and assigned using sequence differences that distinguish them. This requires accurate representation of each copy, reliable subgenome and haplotype labels, and transcript annotations that preserve splice sites and copy identity. Testing should check whether graph-based mapping assigns reads to the correct copies and produces more accurate expression estimates.

The remaining obstacle is not simply the absence of algorithms. Graph-aware aligners cannot be used routinely when species-level graphs, projected transcript annotations, splice-aware indexes, path conventions, and coordinate mappings are unavailable or incompatible. A genomic graph distributed without these components may be technically valid yet unusable for transcriptomics. RNA-seq therefore provides a concrete test of operational readiness: a released graph should support an ordinary user in moving from reads to interpretable expression estimates through a documented and benchmarked workflow. The same principle applies to resequencing, chromatin profiling, epigenomics, and structural-variant genotyping.

## PlantPan as a test of operational readiness

PlantPan, maintained by the National Genomics Data Center and China National Center for Bioinformation, shows both the value and the limitations of centralized graph resources. It integrates chromosome-scale assemblies from 11 plant species with gene groups, genomic variation, synteny, functional annotations, resistance genes, transcription factors, pathways, and homology relationships. Its workflow aligns assemblies to a selected reference with NUCmer (Marçais et al., 2018), detects differences with SyRI (Goel et al., 2019), and incorporates 13 reported variant classes into graphs with VG (PlantPan, 2026). Downloadable graph resources were listed for nine of the 11 species at the time of consultation.

This centralized catalog reduces duplicated whole-genome comparisons and connects non-reference sequences and structural alleles with biological information. Yet the existence of a graph file does not establish a complete graph-reference system. Pairwise discovery against a selected linear reference may retain reference-dependent representations, and graph topology alone does not guarantee stable path identifiers, projected annotations, coordinate mappings, precomputed task-specific indexes, benchmark datasets, release histories, or maintained mapping and genotyping workflows. PlantPan is therefore a valuable foundation and an instructive test case: centralized distribution is feasible, but operational readiness requires additional layers of documentation, indexing, validation, and maintenance.

## A federated release and governance model

No single graph will serve every biological question. A graph optimized for short-read mapping may differ from one designed for comparative annotation, phylogenomics, or visualization. A practical model is a federated family of compatible resources: a stable species backbone, population- or breeding-pool graphs, and focused graphs for complex loci. Shared sample identifiers, path naming, metadata schemas, and coordinate mappings would permit interoperability without forcing all diversity into one monolithic object.

Federation requires governance as well as formats. Each resource needs designated maintainers, persistent release identifiers, transparent procedures for correcting or withdrawing assemblies, compatibility rules, and retained prior versions. Funding agencies should treat these activities as research infrastructure; journals should require deposition of graph packages and build workflows when a pangenome is presented as reusable; and repositories should preserve canonical graphs, metadata, and supported indexes. Graph construction, benchmarking, annotation, and maintenance should be recognized as scholarly contributions.

Access must also be practical. If graph use requires specialized hardware, undocumented pipelines, or transfer of terabyte-scale intermediates, it may widen participation gaps. Projects should offer downloadable canonical files, compact indexes, cloud-ready workflows, representative tutorials, and clear resource estimates. Training should connect graphs to familiar tasks such as read mapping, expression analysis, genome-wide association studies, and candidate-gene inspection.

## Discussion

Plant genomics no longer needs another general demonstration that linear references are incomplete. It needs reusable infrastructure that allows alternative sequence and haplotype diversity to enter everyday analysis. Assemblies document diversity; a graph encodes relationships among alternative sequences; an operational graph-reference package adds the annotations, indexes, coordinate mappings, benchmarks, workflows, release history, and governance required for routine reuse.

The proposed standard can begin modestly. Mature projects should release at least one documented graph with embedded assembly paths, projected annotations, legacy-coordinate mappings, supported indexes, a reproducible build, and held-out benchmarks. Communities can then maintain federated releases for distinct populations and analytical tasks. This proposal complements recent reviews and standards efforts by defining graph publication as an operational commitment rather than a one-time file deposit. Without that commitment, plant pangenomes may remain impressive catalogs used mainly by specialists. With it, their structural and haplotypic diversity can become a routine substrate for plant genomics and breeding.
